# Identification and proteomic analysis of petroleum degrading bacteria

**DOI:** 10.1186/1753-6561-8-S4-P240

**Published:** 2014-10-01

**Authors:** Júlio Nino Souza-Neto, Solange Araújo, Ieda Batista, José Pereira, Edmar Andrade

**Affiliations:** 1 Federal University of Amazonas, Manaus, AM, Brazil; 2 University of the State of Amazonas, AM, Brazil

## Background

The natural environment has suffered high accumulations of pollutants which affect not only the soil, groundwater, rivers, lakes, rainwater routes and air, but also human health due to the formation of unhealthy conditions. Excessive use of pesticides, crude oil spills, discharges of industrial waste, among other xenobiotic compounds accumulate in the environment and become worrisome for its persistence and its harmful effects to life. Therefore, bioremediation becomes an option for solving this problem, which is a tool that uses microorganisms to biochemical pollutants degradation compounds, transforming them into less or non-toxic substances. In this context, this work presents the results of identification of bacteria that degrade crude oil, as well as some results of a proteomic analysis of isolates grown in the presence of that pollutant to evaluate the profile of protein expression.

## Methods

Bacterial strains previously isolated from macrophyte *Eichhornia *sp., *Cyperus *sp. and *Hymenachne *sp., the outputs of Petrobras refinery influent / Manaus-AM (REMAN), were identified by phenotypic analysis and sequencing and comparison of the 16S gene region to identify the species level using the BLAST. Qualitative assays of degradation crude oil were performed for all strains identified using minimum medium and petroleum (1.3%) as carbon source, followed by sample preservation. The sample which had the most degrading activity was selected to proteomic investigation. Growth curve, extraction and quantification of protein content, followed by two-dimensional electrophoresis analysis, were determined for bacteria cultured in presence of 1.3% crude oil or 0.1% yeast extract as carbon source. All assays were performed using proteins extracted at the end of the exponential phase (24h for growth crude oil and 12h for growth yeast extract). The analysis of the spots was made using the Image Master Platinum version 6.0 (GE Healthcare) according to the saliency and smooth parameters, which were equal to 100 and 2, respectively. Proteomic profiles of bacteria cultured in presence of petroleum and yeast extract were compared each other to identify proteins related to crude oil degradation.

## Results and conclusions

Seven samples with the potential for bacterial degradation of crude oil were identified, which are *Pseudomonas aeruginosa *(SB41), *Lysinibacillus fusiformes *(SB63 and SB121), *Acinetobacter junii *(SB102 and SB132) and *Bacillus pumilus *(SB123 and SB139). The species identified showed redundancy and the molecular results were corroborated by phenotype ones. The sample identified as *A. junii *SB132 was used in proteomic analysis which preliminary profiles had been obtained. The number spots identified was 178 and 124 for bacteria cultured in presence of petroleum or yeast extract, respectively. Compared each other protein content for both conditions presented 36% similarity. These data show that the protein profile is different and there is differential expression between the two conditions. Investigation of proteomic profile of other bacterium sample and identification of proteins involved in crude oil degradation are in progress.

